# Jump Horse Safety: Reconciling Public Debate and Australian Thoroughbred Jump Racing Data, 2012–2014

**DOI:** 10.3390/ani5040399

**Published:** 2015-10-22

**Authors:** Karen Ruse, Aidan Davison, Kerry Bridle

**Affiliations:** School of Land and Food, Discipline of Geography and Spatial Sciences, University of Tasmania, PB 78, Hobart, Tasmania 7001, Australia; E-Mails: Aidan.Davison@utas.edu.au (A.D.); Kerry.Bridle@utas.edu.au (K.B.)

**Keywords:** thoroughbred, horse-racing, steeplechase, hurdles, risk, safety, animal-human relationships, media, public debate

## Abstract

**Simple Summary:**

This paper documents the dynamics of Australian thoroughbred jump racing in the 2012, 2013, and 2014 seasons with the aim of informing debate about risks to horses and the future of this activity. We conclude that the safety of Australian jump racing has improved in recent years but that steeplechases are considerably riskier for horses than hurdle races.

**Abstract:**

Thoroughbred jump racing sits in the spotlight of contemporary welfare and ethical debates about horse racing. In Australia, jump racing comprises hurdle and steeplechase races and has ceased in all but two states, Victoria and South Australia. This paper documents the size, geography, composition, and dynamics of Australian jump racing for the 2012, 2013, and 2014 seasons with a focus on debate about risks to horses. We found that the majority of Australian jump racing is regional, based in Victoria, and involves a small group of experienced trainers and jockeys. Australian jump horses are on average 6.4 years of age. The jump career of the majority of horses involves participating in three or less hurdle races and over one season. Almost one quarter of Australian jump horses race only once. There were ten horse fatalities in races over the study period, with an overall fatality rate of 5.1 fatalities per 1000 horses starting in a jump race (0.51%). There was significant disparity between the fatality rate for hurdles, 0.75 fatalities per 1000 starts (0.075%) and steeplechases, 14 fatalities per 1000 starts (1.4%). Safety initiatives introduced by regulators in 2010 appear to have significantly decreased risks to horses in hurdles but have had little or no effect in steeplechases. Our discussion considers these data in light of public controversy, political debate, and industry regulation related to jump horse safety.

## 1. Introduction

Thoroughbred jump racing sits in the spotlight of contemporary welfare and ethical debates about horse racing [1,2]. This activity encompasses hurdling, steeplechasing, point-to-point, and mountain racing and is controversial primarily because it involves higher risk of accident or fatality to both horse and rider compared with flat racing [3,4,5]. Debates about jump racing have been played out via mass media [6,7] and have relevance in ongoing cultural renegotiation of the meanings, norms, and governance of human-animal relationships in modern societies [8,9]. These debates fuse questions about defensible human-animal relations with those about the conduct of human entertainment, sport, and gambling [10]. In particular, falls and fatalities in jump racing are widely reported in the media and result in negative public opinion and criticism of racing and of the racing industry’s approach to equine welfare [2]. Arguably, public concern about horse welfare has contributed to the decline of jump racing in many parts of the world, although economic factors have also been significant [11,12]. Jump racing continues, however, in 18 countries across four continents [11]. While jump racing comprises only a small proportion of horse races and related gambling turnover and prize money, this activity has political, cultural, and economic importance in particular countries, regions and towns [2,11].

Australia provides an important context in which to explore the decline and resilience of jump racing (where it is commonly known as jumps racing). Jump racing is no longer conducted in four of Australia’s six states. However, hurdles and steeplechases remain a feature of thoroughbred racing in Victoria and, to a lesser extent, South Australia. Total annual prize money in Australian jump racing is around $2.7 million (Australian dollars used throughout), with average prize money per race equivalent to that of flat racing [13]. Australian jump racing is a familiar and divisive subject of passionate advocacy and critique in conventional and social media and has been a notable electoral issue in Victorian and South Australian state politics over the past decade [14,15]. In addition to criticism from established animal welfare organisations, such as the Humane Society and Royal Society for the Prevention of Cruelty to Animals (RSPCA), Australian jump racing has been the focus of protest, activism, and lobbying including from groups with a sole focus on horseracing, such as the Coalition for the Protection of Racehorses. 

Table 1 outlines key features of Australian jump racing. Australian jump racing occurs in regional, as well as metropolitan settings in autumn and winter when tracks are softer. Thoroughbreds are specifically bred for jump racing in some countries, but not in Australia where jump horses are drawn from the flat racing population. Australian jump racing is conducted under state-based Local Rules of Racing. There are some differences between the two states: for example, the minimum distance of a hurdle race in South Australia is 2800 m and in Victoria it is 3200 m. In both states, the hurdles in a hurdle race are a maximum of one metre high with horses carrying a minimum weight of 64 kg (jockey and saddle, plus handicap weight). In a steeplechase, horses jump over fences which are at least 1.15 m high. Hurdle races usually involve fewer jumps over shorter distances than steeplechases. In Australia, horses must undergo qualifying trials to be eligible to compete in a hurdle race. Horses may then progress through a sequence from maiden to novice to open hurdle races, before then being eligible to qualify for steeplechase races [16,17]. 

**Table 1:** Key features of Australian jump racing.

**Locations of races**	Restricted to 15 South Australia and Victorian race courses, predominantly non-Metropolitan.
**Type of races**	Two thirds hurdle races, one-third steeplechases.
**Regulatory bodies**	Administered under state-based Local Rules of Racing by Thoroughbred Racing South Australia (TRSA) and Racing Victoria (RVL).
**Industry bodies**	Australian Jumping Racing Association (AJRA).
**Race regulations**	Specify minimum weight (64 kg); course condition rating; height, number and placement of obstacles; maximum field size; use of whips; horse boots; *etc*.
**Industry review**	Seven safety performance reviews by regulatory bodies since 1994.
**Race review**	In addition to the TRSA and VRL Stewards Committee review, TRSA and VRL Jump Review Panels review each horse’s jump at each obstacle in each race and may refer horse or jockey to undergo further training. The Panel includes a former jump jockey who can provide individual coaching if needed.
**Qualification**	Horses, trainers and jockeys must undergo qualification training and trials, overseen by VRL and TRSA, in order to compete in a maiden hurdle race. Horses that progress to open hurdle races are then eligible to qualify for steeplechase races. Mandatory trainer and jockey skills workshops held annually.
**Veterinary inspections**	Of each horse, before and after each race.
**Race season**	March to September. Races are scheduled at approximately fortnightly intervals.
**Number of races**	Less than 100 per season.
**Horses**	Thoroughbreds, drawn from flat racing population, must be at least three years old, and may race in both flat and jump races during the jump race season.
**Tracks**	Left handed turf tracks, no steep downhill runs to finishing-lines.
**Race field**	Field sizes are small, with less than 8 horses on average in a race. Low fields are not uncommon (<5 starters).
**Race start**	Starting gates used at commencement of races.
**Race speed**	Races are run on slow tracks (heavy conditions), with heavier weights carried (>64 kg).
**Hurdle obstacles**	Hurdles are padded panels, maximum 1 metre in height, with standardised design.
**Steeple obstacles**	Steeples are a mix of brush top panels and live hedges not less than 1.15 m in height, depending on race course, with height and width specified by regulator. No water jumps or drops.

During the 2008 and 2009 Australian jump racing seasons, the highly visible deaths of 14 horses in jump races across Victoria and South Australia inflamed criticism by welfare and activist groups, heightened public concern and prompted the prospect of its banning in Victoria [18,19,20]. In late 2008, a group of activist organisations led by Animals Australia presented a submission to the Victorian parliament calling for a ban on jump racing [21]. Their submission reported that 13.1 out of every 1000 horses (1.31%) starting in a jump race (hereafter referred to as “starts”) died [21]. Their submission also summarised longer term fatality rates from 1989 to 2004 based on a 2006 study in the Equine Veterinary Journal documenting the risk of a fatality in Australian jump racing as almost 19 times that in flat racing [22]. This study found that catastrophic limb failure, the predominant cause of horseracing deaths, was approximately 18 times greater for Australian jump racing than flat racing, with cranial or vertebral injury 120 times greater and sudden death 3.5 times greater [22]. Following a further eight deaths in the 2009 jump racing season, Racing Victoria (RVL), the principal authority governing thoroughbred racing in Victoria, commissioned a review of jump racing. The fall and fatality rates for the 2009 season were reported by this review as being 50.8 per 1000 starts and 12.7 per 1000 starts, respectively, the highest recorded during the 2005 to 2009 seasons [23]. These results were despite six previous reviews of horse and jockey safety in Australian jump racing since 1994, with the recommendations of the last of these, the 2008 Jones Report, implemented prior to the 2009 season [21,23]. In November 2009, following their latest review, RVL announced a two year transition plan to phase out jump racing after 2010. However, in January 2010, RVL handed jump racing a tentative reprieve, allowing the sport to continue in 2010 subject to stringent safety conditions and standards including a reduction in fatality rate to approximately half of that for 2009 (*i.e.*, 6.5 per 1000 starts) and a reduction in fall rate to 30 per 1000 starts [24]. The RVL Chairman warned that if the new conditions were not met, jump racing would cease.

In September 2010, following an improvement in the safety of hurdle racing, RVL gave hurdle racing the go ahead for a three year program subject to meeting a key performance indicator (KPI) of a horse fatality rate of not more than 6.5 deaths per 1000 starts (0.65%), measured as a rolling three year average [25]. At the same time, RVL declared the performance of steeplechasing unsatisfactory and requested further measures to improve its safety [25]. However, in October 2010, without the introduction of further measures, RVL agreed to a steeplechase program for 2011, and determined that its future beyond 2011 be subject to a fatality KPI of 6.5 deaths per 1000 starts (0.65%), measured as a rolling two year average [26]. These performance targets were accompanied by a raft of measures to improve horse and rider safety, including the ability of a jockey to withdraw a horse during a race because it is fatigued and out of contention and a danger to itself or the jockey. These initiatives were closely followed in November 2010 by a change of government in Victoria that saw a vocal advocate of jump racing, Dr. Denis Napthine, a veterinarian, installed as Premier and Minister of Racing [27]. With government support, the period 2011 to 2014 saw jump racing authorities in Victoria increase prize money and invest in jump race safety and training infrastructure improvements. By November 2011, RVL discontinued the safety KPIs on the basis that both hurdling and steeplechasing had met their KPIs relating to fatalities for the past two years, undertaking to monitor the safety performance of jump racing on an ongoing basis and to undertake reviews as required [28].

In the context of industry efforts to address concerns about risks to horses in Australian jump racing, advocates argue that horses love to race and jump, that jump racing extends a horse’s career and that many of these animals would be slaughtered if not for jump racing [29,30]. Opponents such as the RSPCA argue that horses have evolved to avoid rather than jump obstacles, that the heightened prospect of injury or death to jump horses is an unacceptable focus of human entertainment, and that the risk of being injured or killed in jump racing is not an acceptable alternative to the slaughterhouse [31,32,33,34]. The Humane Society describes jump racing as institutionalised cruelty [35].

Recent debate about Australian jump racing has taken place in the absence of sufficient robust or current data, with opponents continuing to rely on Boden *et al.*’s 2006 study [32,34,35,36]. The composition and dynamics of the cohorts of jockeys, trainers and horses involved in Australian jump racing, the ages and career trajectories of jump horses, the geography of this activity, and annual changes in activity have not been reliably documented nor related to data about horse safety. In response, this paper compiles, synthesises, and analyses data collected by Racing Australia (RA, formerly Racing Information Services Australia), for the 2012, 2013, and 2014 jump race seasons in Victoria and South Australia. In addition to informing public and policy debate, the paper contributes to international understanding of the dynamics of Australian jump racing in the context of changes in the horse racing industry more generally, including changing attitudes on questions about the use of animals in public entertainment. 

## 2. Methodology

Jump race data (hurdle and steeplechase) were obtained from RA for the 2012, 2013, and 2014 racing seasons from the Results area of the RA website. Although publicly available, these data are recorded on a race-by-race basis and are not aggregated, nor are composite trends identified. Data retrieved included; race name, location, date, time, distance and course condition rating, as well the name of each horse, jockey, and trainer involved in each race. Seasonal data on total starts for the period 2007 to 2010 were obtained from the Australian Racing Fact Book. Every thoroughbred race in Australia is reviewed by an official Stewards Panel which monitors racing conduct and injuries to horses. For jump races, a Jump Review Panel (JRP) subsequently reviews how well each horse jumped each obstacle. Stewards’ reports were obtained from RA and JRP reports from RVL. Although a similar panel reviews South Australian jump races these reports are not publicly available. 

Data were entered into a Microsoft Excel ^TM^ (2013) database and ordered in the form of a “start”, or an individual horse leaving a starting gate in a jump race. Data were organised by racing season, which extends from March to September of a calendar year. Annual data thus relate to a single jump race season, except for data in Figure 4 which has been standardised to an annual racing year (1 July to 30 June) to allow for comparison with datasets from other studies. Horses listed in a jump race may be “scratched” before a race due to a variety of reasons, for example, disqualification by a veterinarian. Scratched horses are not included in the database. The database records individual horse performances and race placings and lists intra-race incidents in each race from the official Stewards reports, including, falls, run outs, lost rider, brought down, and failed to finish (a term used in official race reports to describe a horse withdrawn during a race at the discretion of the jockey). Jump Review Panel Reports about horse performance at each jump were matched to the official Stewards Reports for each horse, including details of fatal falls and other race incidents. Our database therefore provides a comprehensive picture of individual horse performances as well as a means to aggregate Australian jump race information over the study period. Our initial purpose was to uniquely identify jump horses, their trainers, and jockeys in order to describe the size, scope, and location of Australian jump racing and risks to horses over this period.

Starts were summed by horse by the state they raced in. The number of individual horses participating over this period was calculated by aggregating starts against horses’ names and uniquely identifying each horse based on the RA horse search. The home state of each horse and trainer was identified by matching horses to trainers and identifying the trainer’s place of residence from the addresses shown for official qualified trainers. The information generated included starts per state, as well as the number of starts by each trainer. Our analysis also identified horses that only raced in hurdles, horses that competed in hurdles and steeplechases, and those who competed only in steeplechases. 

Horse falls and fatality rates were calculated by dividing the number of falls and fatalities by the total number of starts in all races, for each season and in the overall sample. Only race fatalities were included in the analysis; training and trial fatalities were not considered. The average number of starts in each race was calculated by dividing the total number of jump race starts by the total number of races. A trainer operating both in a partnership and also in their own name was counted as two separate entries.

## 3. Results

### 3.1. Location of Australian Jump Racing

**Figure 1:**
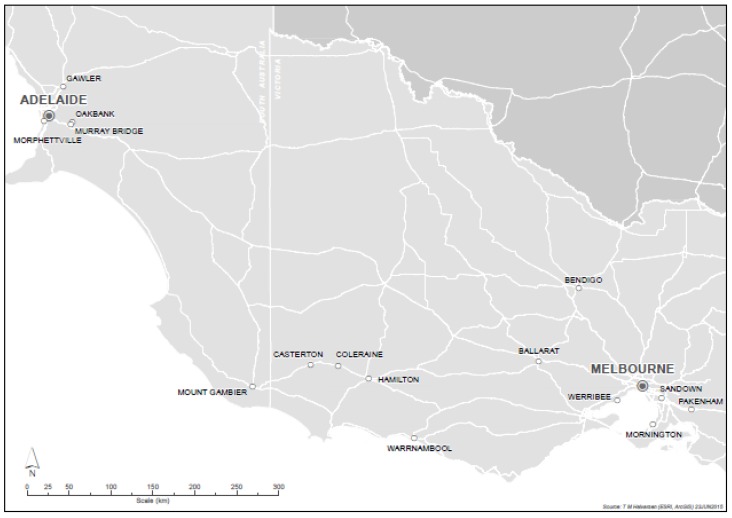
Location of Australian jump racing clubs, South Australia and Victoria. Clubs hosting jump races over the period 2012 to 2014.

The study encompassed 257 jump races, comprising 171 hurdle races and 86 steeplechases, conducted in Victoria and South Australia over the 2012, 2013, and 2014 jump race seasons. This represents less than 1.5% of total thoroughbred races in South Australia and Victoria over this period [37]. The majority of jump races (67%) were held in Victoria. Only 15 of the 386 racing clubs in Australia conducted jump racing; five in South Australia and 10 in Victoria [37]. The distribution of clubs is shown in Figure 1. 

Australian jump racing is concentrated in western Victorian country (rural) racing clubs (Figure 1). Warrnambool Racing Club hosted 28.4% of jump races over the study period, more than any other location, providing the hub for the Australian jump racing industry. The Warrnambool Racing Club’s May carnival attracts around 30,000 people and includes Australia’s longest jump race, the Grand Annual Steeplechase (total prize money t.p.m. $250,000), over 5.5 km and 33 jumps (4). Only two metropolitan clubs held jump races, although they accounted for 26% of races over the study period. The South Australian Jockey Club at Morphettville, Adelaide, held 44% of South Australian jump races, and 10.1% of all jump races. The Melbourne Racing Club at Sandown, Melbourne, hosted 21.2% of Victoria’s jump races, and 16.3% of all jump races, including the Grand National Hurdle (t.p.m. $200,000) and Grand National Steeple (t.p.m. $250,000).

### 3.2. Participants in Australian Jump Racing

#### 3.2.1. Horses

Over the 2012, 2013, and 2014 seasons, 438 individual horses participated in 1970 jump race starts. In keeping with the proportion of hurdle to steeplechase races, over two thirds of jump horses (302, 69%) competed only in hurdle races; just under one quarter (99, 23%) competed in both forms of Australian jump racing; and 37 (8%) competed only in steeplechases. 

**Figure 2:**
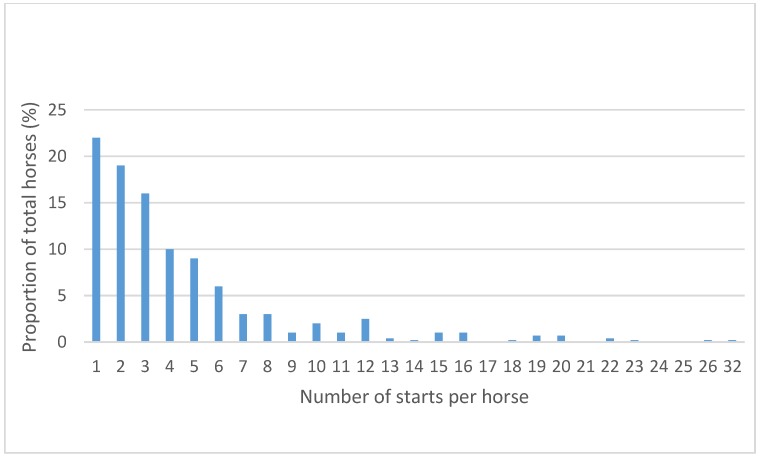
Proportion of horses (%) by number of starts per horse over the 2012, 2013, and 2014 seasons, South Australia and Victoria combined.

Figure 2 shows the number of starts per horse over the study period. More than half of horses (55%) competed three times or less, with almost one quarter (22%) competing in only one race. Another quarter raced between four and 10 times. Less than 10% of horses competed more than 10 times, with one horse racing 32 times. The median number of starts per horse was three, the first quartile was two starts and the fourth quartile was six starts. The range was one to 32 starts.

Annual turnover in the jump horse cohort was analysed by identifying individual horses that ran in two of the three race seasons, either in successive seasons or in 2012 and 2014, or in all three seasons (Table 2). Of the 2012 jump horse cohort, 37% raced in 2013 and 21% raced in 2014. Only 7% of the 2012 cohort raced in 2014 but not in 2013, and only 14% raced in all three seasons. Of the 2013 cohort, 29% raced in 2014.

**Table 2:** Australian jump horse annual turnover from the 2012 to the 2013 and 2014 seasons.

Number of Horses 2012	Number of Horses 2013	Number of Horses 2014	Number of Horses 2012 & 2013	Number of Horses 2012 & 2014	Number of Horses 2012, 2013 & 2014	Number of Horses 2012 & 2014 but not 2013
176	209	195	65 (37%)	40 (21%)	27 (14%)	13 (7%)

Number of horses that jump by season(s) of participation. All % figures indicate a proportion of the 2012 jump horse cohort. Horses are counted in the cohort of each season in which they competed.

**Figure 3:**
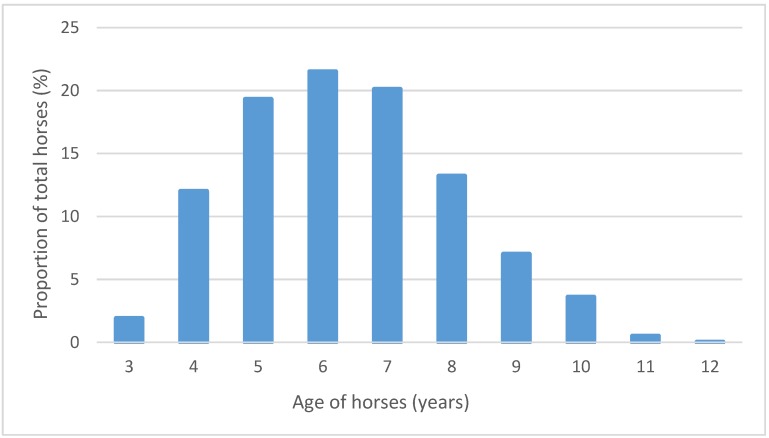
Age profile of jump horses, as a proportion of all horses jumping during the 2012, 2013, and 2014 seasons. The age census date is the beginning of the jump racing season (1 March) of each calendar year in which a horse competed. The total horse pool is the sum of the 2012, 2013, and 2014 horse cohorts, and not the number of individual horses over this period.

The average age of horses in the sample was 6.4 years, as of 1 March in the seasons in which they raced in the 2012–2014 time period. Median age was six years, the first quartile median age was five years and the fourth quartile median age was seven years. (Figure 3). Consistent with the regulatory requirement that a horse be at least three years old to begin jump training and racing, and the time required for this training, just eight horses (2%) in our sample were aged three years in their first season of jump racing. Twenty five horses (7%) were aged 10 years or more. The range was three to 12 years. 

#### 3.2.2. Trainers

In 2014, there were 4027 registered race horse trainers in Australia, with 914 in Victoria and 304 in South Australia (5). Only 145 trainers, less than 4% of the total, started a horse in a jump race between 2012 and 2014 (Table 3). Three quarters (76%) of these trainers were based in Victoria (110), with 31 (21%) based in South Australia, one in New South Wales and three in New Zealand. Victorian trainers account for the majority of starts in jump racing. A small number of prominent Victorian trainers account for a disproportionately large share of activity. Table 3 lists the five most prolific trainers in Victoria and South Australia, who accounted for 34% and 6%, respectively, of jump starts in the study period. Five trainers (two Victorian, two South Australian, and one New Zealander) accounted for half of the starts in South Australia, and the majority of horses in South Australian jump races were trained in Victoria. The most prolific Victorian trainer accounted for 20% of all starts in South Australia.

**Table 3:** Trainer participation by location in Australian jump racing, 2012–2014.

Trainer Rank	Number of Starts	Proportion of Total Starts (%)	Proportion of Victorian Starts (%)	Proportion of South Australian Starts (%)	Number of Horses Trained
**Victoria**					
1	231	11.7	9.2	19.9	34
2	136	6.9	7.7	4.4	21
3	135	6.9	7.3	5.5	24
4	89	4.5	5.1	2.8	25
5	79	4.0	4.8	1.5	18
**South Australia**					
1	67	3.5	1.0	12.1	15
2	32	1.6	0.1	6.6	3
3	10	0.5	0.2	1.5	6
4	7	0.3	n/a	1.5	3
5	7	0.3	n/a	1.5	3
**New South Wales**					
1	4	0.4	0.5	0	4
**New Zealand**					
1	33	1.7	0.6	5.2	12
2	3	0.15	n/a	0.4	2
3	1	n/a	n/a	0.2	1

Top trainers are ranked by number of starts. Only the 5 top trainers for Victoria and South Australia are listed.

#### 3.2.3. Jockeys

Relative to horses (438) and trainers (145), the cohort of jump jockeys in the study period was small, at 51. Around 30 jockeys rode in all three Australian jump racing seasons, with the majority moving frequently between South Australia and Victoria. Ten jockeys accounted for 62.1% of starts, with three jockeys accounting for 25.3% of starts. The most prolific jockey rode in almost four out of every five (77%) jump races over this period.

### 3.3. Horse Falls and Fatalities

Table 4 shows the fall, fatality, and finish rates for jump horses in the study period. The overall fatality rate was 5.1 per 1000 starts (0.51%). The overall fall rate was 33 per 1000 starts (3.3%). Around 10% of all jump horse starters were retired before the race finished, falling into the category “failed to finish”. Other reasons for not completing a race include “lost rider” (*i.e.*, jockey falling off) (1.6%), “brought down” (*i.e.*, horse brought down by another horse’s fall) (0.35%) and, in one case, “run out” (*i.e.*, horse jumped out of the race course). Overall, 85% of horses starting a jump race completed the race. 

A significant difference in risk profile was evident between steeplechase and hurdle races during the study period (Table 4). Of 10 horse fatalities, nine occurred in steeplechases, with the single hurdle fatality occurring on the flat at the start of a race, rather than over a hurdle. This disparity is increased when the larger proportion of hurdle races is taken into account, with one fatality in 1328 hurdle starts compared to nine fatalities in 642 steeplechase starts. The fatality rate for steeplechases was 14.0 per 1000 starts, more than double the KPI; and the rate of 0.75 per 1,000 starts for hurdles, was almost an order of magnitude below the KPI. This disparity is less marked when considering the rate of falls in hurdles (29 per 1000 starts) and steeplechases (40 per 1000 starts). However, the proportion of falls that result in fatalities in steeplechases (35%) is more than an order of magnitude greater than for hurdles (2.6%), with over one third of steeplechase falls proving fatal.

**Table 4:** Hurdle and steeplechase horse falls, fatalities and finishes, Victoria and South Australia, for the 2012, 2013, and 2014 seasons.

Race Type	Starts	Finishes	Deaths	Fatality Rate (Deaths per 1000 Starts)	Falls	Fall Rate (% of Starts)	Fatalities as Proportion of Falls (%)	FF*	BD**	RO***	LR****
**Hurdle**	1328	1135	1	0.75	39	2.9	2.6	128	7	1	18
**Steeplechase**	642	537	9	14	26	4.0	35	65			14
**Total**	1970	1672	10	5.1	65	3.3	15	193	7	1	32

* Failed to Finish (FF): horse withdrawn during race as fatigued and uncompetitive at discretion of jockey; ** Brought Down (BD): horse brought down during race by another horse; *** Run Out (RO): horse leaves track during race; **** Lost Rider (LR): jockey falls from horse during race.

**Figure 4:**
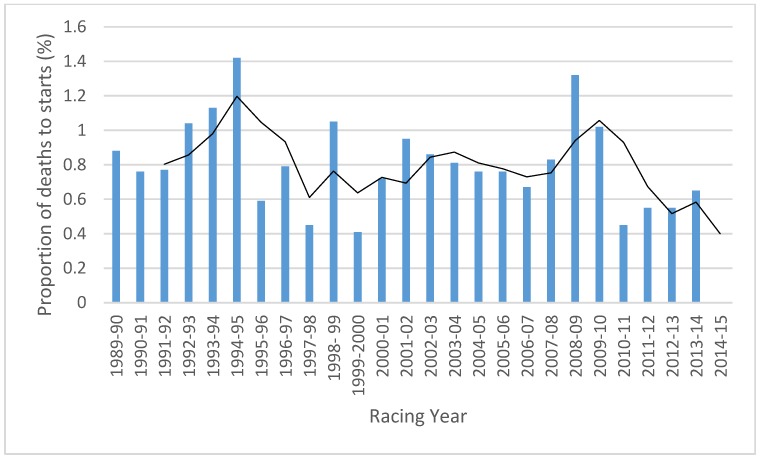
Horse fatality rates in Victorian jump racing, 1986 to 2015. Source data: Boden *et al.* 2006 [22], Australian Racing Fact Book 2013 [37], and the Animals Australia submission to the Victorian Parliament 2008 [21]. A racing year is defined as the period 1 July to 30 June in the following year. Fatality rates for the period 2011–2014 rates were estimated by identifying the date of the fatality and aggregating deaths over the racing year. Data for 2004–2005 and 2005–2006 are average values sourced from the 2008 Animals Australia submission to Victorian MPs as individual fatalities could not be located for this period [21].The line represents the moving three year average.

We compared our study period against longer term trends using publicly available data for Victoria (comparable data were not available for South Australia). The resulting data (Figure 4) indicate that annual Victorian fatality rates during the study period are lower than any other consecutive three year period since 1986. These rates show considerable variability, with the lowest annual fatality rate in our study period, 5.5 deaths per 1000 starts in 2011–2012 and 2012–2013; the lowest annual rate being 4.1 deaths per 1000 starts (0.41%) in 1999–2000 (and 4.5 deaths per 1000 starts (0.45%) in 1997–1998. 

## 4. Discussion

Current public debate about Australian jump racing is taking place in the absence of comprehensive and reliable data and is characterised by competing claims by supporters and opponents. We use the data presented above to assess a range of contentious issues related to risks to horses in Australian jump racing. 

### 4.1. Risks to Horses in Hurdles and Steeplechases

There is currently no horse fatality rate target mandated in Australian jump racing [16,28]. However, our data show that the fatality rate across the 2012, 2013, and 2014 jump racing seasons in Victoria and South Australia combined, 5.1 deaths per 1000 starts (0.51%), is below the Victorian industry KPI of 6.5 deaths per 1000 starts (0.65%) set in 2010 (and discontinued in 2011). The fatality rate observed in any one season of the study period is below half of that in the 2008, 2009, and 2011 seasons, and no more than three quarters of the rate in 2010 (Figure 4). This indicates that, despite the removal of mandated KPIs, safety initiatives introduced by RVL in 2010, and also adopted by South Australia, have had some success in reducing fatalities. These initiatives included improving the placement of obstacles, improving schooling and trialing facilities, and assessing the suitability of venues to conduct steeplechase races [12,26,28,38]. In particular, our data show that a new rule in 2010, allowing jockeys to retire a horse during a race if fatigued and out of contention, was invoked for about 10% of starts. Given that fatigued horses are more likely to pose a risk to themselves, their rider, or other horses, frequent application of this rule is likely to have been a significant contributor to improved horse and human safety in the study period [39,40,41]. Our finding that just 10 jockeys accounted for almost two thirds of jump race starts in the study period, raises the question also of whether improved safety performance is linked to the presence of a small, highly-experienced cohort of jump jockeys. Previous research in the United Kingdom supports jockey experience and consistent pairing of horse and jockey as key variables in reducing the risk of falls [42,43]. While the pool of Australian jump trainers is almost three times that of jockeys, this activity is also concentrated within a small, highly-experienced group, with five Victorian trainers accounting for one third of starts in Victoria and South Australia (Table 3).

To regulators and advocates of Australian jump racing, recent reductions in horse deaths indicate a laudable and sustainable improvement in horse welfare [44,45]. However, our data also indicate that horses continue to die in jump races. In addition to the risk of catastrophic injuries associated with falls over jumps [22,46], it has been suggested that an older horse population and longer race distances may contribute to a higher intrinsic risk in jump racing, compared to flat racing [47]. To opponents of Australian jump racing, then, jump racing cannot be run safely or humanely [31,32,33,34,48] with unjustifiable and avoidable horse deaths during jump races each season being predictable; “you are just waiting for it (a fall) to happen” [33]. Reflecting this, each horse death during the study period attracted media attention, with activist groups calling for an immediate ban on Australian jump racing, raising petitions and stating their intention to mount public protests at every jump race trial and race meeting in Victoria [48,49]. Horse injury and death in training and trials were not included in industry reporting of Australian jump racing fatality rates in our study period. A complete picture of horse fatalities in Australian jump racing would need to include training and trials as well as races. In 2015, RVL strengthened their Local Rules of Racing to require the death of a horse in training (including trials) to be reported by the trainer [17]. While our data show that industry responses to concern about horse welfare in Australian jump racing have reduced risks over the period 2012–2014, considering hurdle and steeplechase races separately reveals a disparity rarely raised in debate about Australian jump racing. No horse died while jumping a hurdle in a race in the study period, despite the fact that there were 39 falls during hurdle races. The rate of 0.75 fatalities per 1000 starts for the study period is considerably lower than that previously documented for hurdle races (6.3 fatalities per 1000 starts), suggesting that the current design and placing of hurdles is effective in reducing risks to horses during falls [22]. Steeplechase races, however, were riskier for horses, with nine of 26 falls over fences resulting in a fatality, despite the introduction of significant safety measures prior to the study period. The steeplechase fatality rate in the study period of 14.0 fatalities per 1000 starts (1.40%) is comparable to that documented by Bailey *et al.* of 14.3 [50], and higher than that documented by Bourke of 11.0 [51]. While the higher obstacles of steeplechases are likely to contribute to increased fall rates, the presence of more obstacles over longer distances, compared to hurdle races, enhances the risks of the onset of fatigue and the related risks of falling. In the United Kingdom, the risks of falling and/or injury were associated with greater race speed [40,41,46,52], race distance [53], fence type and location [40,41,53], going or track condition [54], number of runners [53,54], and experience of the horse [40] or jockey [41,54]. Fall risk increased in races with over thirty runners [42,43], greater race speed, especially in the second half of the race, races run at a faster pace [39,40,41,53] and longer races [42]. Over 90% of falls in UK jump racing were associated with horses colliding with an obstacle [40]. 

While steeplechases comprised only one third of jump races during the study period, and around 30 races in any one season, each of the 10 horse deaths occasioned in these races generated considerable negative media attention, energised anti-racing activists, and increased political pressure on the thoroughbred racing industry more generally [31,32,33,34]. However neither the RSPCA (South Australia and Victoria) nor the Coalition for the Protection of Racehorses distinguish between the inherent risk of hurdle races and steeplechases. While the Coalition for the Protection of Racehorses drew attention to a peak in hurdle deaths in 2009, they continue to combine data for steeplechase and hurdles in their critique, claiming that the statistics are so variable from year to year that there is no clear cut answer to whether or not steeplechases are riskier than hurdle races [33]. 

Supporters of Australian jump racing have not sought to respond to negative publicity about jump racing by drawing attention to the recent improved safety record of hurdle racing, the dominant form of Australian jump racing. The lack of any fatality over a hurdle in the study period, and an overall hurdle race fatality rate close to that of flat racing could potentially be used to question the assumption of critics that jump racing is inevitably far riskier than flat racing. Certainly, steeplechase races are among the most prestigious in Australian jump racing and include the two most lucrative jump races (in terms of prize money), with the average prize pool of steeplechases and hurdles in 2015 in Victoria being $64,000 and $35,000, respectively [29]. In the context of the status of steeplechase racing in Australian jump racing, future research to understand why safety interventions that appear to have successfully decreased the risk of hurdle races have not realised a similar reduction in risk for steeplechases seems warranted. In particular, the risk associated with specific race tracks is an area worthy of further study given the work of Williams *et al.* (2013) about risk factors for falls and fatalities associated with individual race courses [4,5,39]. Objective risk analysis and better understanding of sources of risk that result in falls and fatalities may ultimately result in better targeted risk mitigation strategies with a consequent reduction in falls, injuries and fatalities to both horses and jockeys [55]. A multi-disciplinary agenda for research that could reduce accident, injury, and death through risk mitigation strategies, as suggested by Thompson, McGreevy, and McManus, appears warranted [56]. 

Conflicting perceptions of acceptable risk, and the diverse value-judgments on which these may be based, are at the heart of debate about Australian jump racing. Typically those horse sports where there is a higher risk of injury or death to the horse, for example, bull fighting, rodeo, chuck wagon racing, and the cross country component of eventing, attract adverse media commentary, controversy, and public debate about horse deaths and the use of horses for public entertainment [6,57,58,59]. Horse deaths in jump races are highly public and often involve spectacular falls, providing activist groups with graphic photographs. The widespread use of such images on social media increases the visibility and awareness of the risks of Australian jump racing. Increased visibility is an advantage in promoting improved welfare outcomes, and a larger volume of protective legislation is generated in the case of animals with a high level of visibility. As O’Sullivan argues, “the community needs to know and like an animal for that animal to have a chance of receiving effective legal protection” [60]. Equally, the highly public and inherent risk involved in Australian jump racing may well be integral to the attraction this activity holds to those who view it as “the thrill of the chase” and an entertainment comprised of courageous horses, hardened trainers, fearless riders, and controlled danger in “the greatest show on earth” [61,62] .

### 4.2. Does Jump Racing Extend a Horse’s Racing Career?

Unlike those in the UK and Ireland, Australian jump horses are not selectively bred for jumping, and are usually former flat racing horses bred to run longer distances. The progression from flat to jump racing in Australia is the basis of claims from advocates that jump racing extends a horse’s racing career, giving them “a new lease on life”; not just by extending their tenure within the industry, but also by renewing a horse’s desire for racing [63,64]. In Australia, rules of racing mandate that a horse has to be at least three years old before it can commence jumping. Given the requirements to qualify a horse to jump, it is likely that the majority of Australian jump horses will be at least four years old before racing over obstacles. Our data show the median age of jump horses is six years, with a range of three to 13 years and almost one quarter aged eight and above (Figure 3). 

The anti-jump racing organisation, the Coalition for the Protection of Racehorses, has argued that Australian race horses are separated from their mothers at about six months of age to commence preparation for sale and training and have an “average career of … less than three years,” after which “the majority will be killed” [33,34]. If this claim has merit, our finding that the median age of a jump horse is six years, with almost one quarter aged eight and above, indicates that Australian jump racing may well significantly extend the tenure of some horses within the racing industry. Given that jump racing accounted for only 1.5% of all thoroughbred racing in Victoria and South Australia the study period, this career option will be offered only to a small minority of race horses.

Our data about the age profile of jump horses needs to be understood, however, in the context of a high rate of annual turnover in the jump horse cohort. Despite the investment in training and qualifying a jump horse, 55% of horses started in three or fewer races across the 2012, 2013, and 2014 seasons, with 22% starting in only one race (Table 2). Only 37% of the 2012 cohort jumped in 2013, while only 29% of the 2013 cohort raced in 2014. Less than 5% of the 2012 cohort participated in all three seasons. The career of the majority of jump horses thus involves a small number of hurdle races in a single season of racing. This finding is consistent with the pattern of jump racing in New Zealand where jump horses have fewer starts than flat racing thoroughbred horses and represent an older horse population [65]. Many flat racing careers are also of limited duration and may also consist of a limited number of starts [66]. This suggests that any career extension enabled by Australian jump racing is short lived for the majority of horses. It also suggests that, given a median jump horse age of six years, the flat careers of jump horses either started later or progressed for longer than claimed by Coalition for the Protection of Racehorses. It is also clear that Australian jump racing is sustained by high levels of new horse entries each season. This high rate of turnover raises questions about horse pathways in and out of Australian jump racing. These questions are relevant to public debate about the drivers of horse breeding in Australian racing [11,67]. 

### 4.3. The Future of Jump Racing

Much opposition to Australian jump racing has called for the abolition of this activity, a prospect that seemed imminent in Victoria in 2010 [20]. In response to these calls, many advocates point to the vital role of jump racing within the Australian racing industry as a whole and within specific regional economies and cultural identities [7,45,68,69]. Recent media commentary suggests that Australian jump racing has enjoyed a resurgence following the election of the pro-jump Napthine State Government in 2011 [45,69]. Our study shows no evidence of either significant decline or significant growth in Australian jump racing over the period 2012 to 2014. However, in 2015, the South Australian Jockey Club expressed a wish to phase out jump racing at Morphettville. The Minster for Racing, Leon Bignell, stated his desire to end the activity; a parliamentary committee has been set up to investigate the future for jump racing; and a member of State Parliament, the Greens MLA, Tammy Franks, has introduced a bill to ban the activity [14,70]. 

The extent of public controversy about Australian jump racing might suggest that this activity is widespread and substantial. Our data, however, indicates that geographically, and in terms of the human participant base, this activity is highly concentrated. Three race tracks—Morphettville (Adelaide), Sandown (Melbourne) and Warrnambool (South-West Victoria)—accounted for over half (55%) of this activity, with South-West Victoria its vital heartland. Three trainers in Victoria trained one in four of all jump horses in Australia, including almost one in three in South Australia. Three jockeys accounted for over one quarter of all jump race starts (25.3%), with each participating in a majority of races. These data indicate that the presence of jump racing in South Australia is highly dependent upon participation from the Victorian racing industry. Any reduction or banning of jump racing in South Australia will have some impact on the Victorian industry. The banning of jump racing in Victoria would likely mean the end of this activity in Australia. While an end to Australian jump racing might affect only a relatively small number of industry livelihoods, racing clubs, and regional communities, the extent of this impact on these individuals, groups and places will be profound. It is not surprising, therefore, that the defence of Australian jump racing is vociferous, particularly in regional Southwest Victoria [68]. The Warrnambool Racing Club’s May carnival, for example, attracts an audience equivalent to the entire population of this regional centre [68]. This event is regarded locally as a major boost for regional tourism and local businesses. “After all, the three day carnival is said to be worth $15 million to the town (Warrnambool). Pubs and restaurants swell in an annual blur of heartiness and hangovers. ‘If we lose it the town is screwed,’ says one well-placed observer” (p. 20, [71]). Similarly, in South Australia, the iconic Oakbank Easter racing carnival is reputably worth $13 million to the local economy and draws crowds around 70,000 to the two days of jump racing [70].

It is too early to assess the implications for Australian jump racing of a change of government in Victoria in late-2014, although the new Minister for Racing has said that the future of jump racing rests in the hands of RVL [72]. It is noteworthy that the 2015 Victorian jump racing season is being conducted entirely at regional race courses, and not at Sandown in Melbourne, a venue that accounted for 16.1% of all jump races between 2012 and 2014 [73]. In light of on-going public debate about Australian jump racing, longitudinal, industry-wide and composite data, such as that presented here, is vital for informed discussion and effective regulation. We note that while the data on which this paper is based are publicly available, and are also highly fragmented and dispersed, requiring considerable effort to assemble and integrate. We recommend that racing authorities consider forms of data collection, recording and archiving that are more amenable to analysis of industry-wide and long-term trends. Reasons underlying short-lived thoroughbred jump racing careers also deserve further research, particularly given the significantly greater investment in time required to train and qualify a jump horse in Australia.

## 5. Conclusions

We conclude that the safety of Australian jump racing over the 2012, 2013, and 2014 seasons has improved significantly from the 2008 and 2009 seasons that provoked strong public opposition and led regulators to canvass the banning of this activity. However, it is not clear that there has been any improvement in horse safety in steeplechasing, with this activity accounting for nine of the 10 horse deaths in the study period. The risks to horses in hurdle racing during the study period, in contrast, were close to those documented for flat racing. While the average age of Australian jump horses indicates that jump racing may extend their tenure within the racing industry, the jump racing career of a majority of horses comprises no more than three races conducted in one season. Jump racing in South Australia is substantially dependent upon Victorian involvement and, overall, Australian jump racing relies upon significant participation from a very small number of trainers and jockeys. 
